# Trends in Treatment and Perioperative Outcomes of Upper Tract Urothelial Carcinoma: The Evolving Role of Lymphadenectomy and Neoadjuvant Chemotherapy

**DOI:** 10.3390/jcm15072536

**Published:** 2026-03-26

**Authors:** Robert Bischoff, Nikolaos Pyrgidis, Benedikt Ebner, Yannic Volz, Julian Hermans, Marie Semmler, Patrick Keller, Gerald B. Schulz, Julian Marcon, Philipp Weinhold, Christian G. Stief, Lennert Eismann

**Affiliations:** Department of Urology, University Hospital Munich, Ludwig Maximilian University of Munich, Marchioninistr. 15, 81377 Munich, Germany

**Keywords:** UTUC, radical nephroureterectomy, nephron-sparing surgery, neoadjuvant chemotherapy, lymphadenectomy

## Abstract

**Objectives:** Real-world data on surgical and multimodal management of upper tract urothelial carcinoma (UTUC) are limited. This study examined epidemiological trends, nephron-sparing surgery adoption, and the perioperative impact of lymphadenectomy (LND) and neoadjuvant chemotherapy (NAC). **Methods:** The German Nationwide Inpatient Data (GRAND) registry (2005–2023) identified UTUC patients undergoing radical nephroureterectomy (RNU), endoscopic laser destruction, or segmental ureteral resection (SUR) using OPS codes. Demographics, comorbidities, complications, and in-hospital mortality were extracted from ICD-10-GM data. Multivariable regression adjusted for baseline comorbidities assessed associations between treatment type, LND, NAC, and perioperative outcomes. **Results:** Among 53,427 UTUC patients, 77.3% underwent RNU, 13.8% endoscopic laser destruction, and 8.9% SUR. Endoscopic laser use rose from <10% (2005) to about 20% (2023). LND was performed in 13% of RNU cases, increasing from 1.1% to 19%. LND was associated with higher risks of transfusion (OR 1.47, 95% CI 1.37–1.57), acute kidney injury (OR 1.19, 95% CI 1.07–1.32), and ICU admission (OR 1.21, 95% CI 1.13–1.30), without affecting in-hospital mortality. NAC was given to 1.7% of patients, with a five-fold increase over time, and was associated with more transfusions (OR 1.28, 95% CI 1.07–1.52) and urinomas (OR 2.31, 95% CI 1.31–3.78), but not mortality. **Conclusions:** UTUC management is evolving, with growing use of endoscopic laser therapy and guideline-aligned lymphadenectomy during nephroureterectomy. Neoadjuvant chemotherapy remains underused despite acceptable perioperative safety, highlighting the need for increased awareness to optimize multimodal treatment.

## 1. Introduction

Upper tract urothelial carcinoma (UTUC) accounts for 5–10% of all urothelial carcinomas and is associated with poor oncological prognosis and impaired functional outcomes [1,2]. In contrast to urothelial carcinoma of the bladder (UCB), UTUC frequently presents with invasive, high-grade disease at diagnosis, making radical nephroureterectomy (RNU) the standard treatment in most cases. However, a subset of patients with low-risk UTUC—characterized by low-grade histology, unifocal tumors, and non-invasive growth— benefit from organ-preserving approaches such as endoscopic laser ablation [3].

Over the past decade, the clinical management of UTUC has evolved due to earlier detection through widespread use of cross-sectional imaging and advancements in endoscopic technologies [4,5]. These developments have expanded the role of kidney-sparing surgeries (KSS) in low-risk and carefully selected high-risk patients. Simultaneously, growing evidence supports the integration of systemic therapies in high-risk UTUC. Notably, the POUT trial demonstrated improved survival with adjuvant platinum-based chemotherapy in patients with invasive disease or nodal involvement [6]. Neoadjuvant chemotherapy is increasingly considered, especially in anticipation of renal function decline after RNU, though prospective data remain limited [7,8]. The role of template-based lymphadenectomy, meanwhile, continues to be debated, particularly regarding its oncological benefit and associated perioperative risks [9].

Historically, UTUC treatment strategies were largely extrapolated from UCB management. However, recent evidence has prompted the development of UTUC-specific clinical guidelines, reflecting a shift toward more individualized care pathways. Despite these advancements, it remains unclear how these changes have influenced real-world clinical practice at the population level.

To address this gap, we conducted a nationwide retrospective cohort study using the German Nationwide Inpatient Data (GRAND) registry, encompassing all UTUC patients treated in Germany over the past 19 years. Our primary aim was to investigate temporal trends in therapeutic strategies, with secondary objectives to evaluate the utilization of neoadjuvant chemotherapy, lymphadenectomy, and associated perioperative complication rates.

## 2. Patients and Methods

### 2.1. Nationwide Inpatient Dataset (GRAND)

This study utilized data from the German Nationwide Inpatient Data (GRAND) registry, maintained by the Federal Statistical Office of Germany (Wiesbaden, Germany), as previously described [10,11]. The GRAND database covers all inpatient hospitalizations across Germany—including public and private institutions—since 2005, excluding military, psychiatric, and forensic hospitals. Data access was granted under project number LMU-4710-2022.

Under the German Diagnosis-Related Groups (G-DRG) reimbursement framework, all hospitals are required to report inpatient diagnoses and procedures to the national database. Clinical diagnoses and perioperative events are coded according to the International Statistical Classification of Diseases and Related Health Problems, 10th revision, German Modification (ICD-10-GM), and surgical procedures are documented using the Operationen- und Prozedurenschlüssel (OPS) system. Codes for RNU were 5-554.5; 5-554.b, for endoscopic laser therapy 5-552.6; and 5-563.4, for SUR 5-563.0. Coding practices are standardized by national guidelines of the German Institute for Medical Documentation and Information. Outpatient procedures (i.e., same-day discharges) are not included, and follow-up is limited to the index hospitalization.

### 2.2. Study Population and Variables

We identified all hospitalized patients who underwent surgical treatment for upper tract urothelial carcinoma (UTUC) between 2005 and 2023. Procedures were classified using specific OPS codes for radical nephroureterectomy, endoscopic laser destruction, and segmental ureteral resection (SUR). Baseline demographics, comorbidities, and in-hospital complications were extracted using ICD-10-GM and OPS codes (Supplementary Tables). The primary endpoint was the temporal assessment of surgical approaches for UTUC. Secondary endpoints included perioperative morbidity (sepsis, acute kidney disease, embolism, transfusion, ileus, urinoma, and ICU admission), in-hospital mortality, and length of stay. Additional analyses focused on the use of lymphadenectomy and neoadjuvant chemotherapy in RNU patients.

### 2.3. Data Processing and Statistical Approach

Data were accessed in anonymized form through the Research Data Center of the Federal Statistical Office. In compliance with German data protection law, individual-level data were not available to the investigators; thus, ethical approval and patient consent were not required. All analyses were conducted by the Research Data Center using RStudio (Version 2025.09.1+401 (2025.09.1+401)) scripts provided by the study team (source: DRG Statistics 2005–2023).

To maintain confidentiality, results for patient groups with fewer than three individuals per category were censored.

Categorical variables are reported as counts and percentages, and continuous variables as medians with interquartile ranges (IQR). Multivariable logistic and linear regression models were applied to evaluate associations between treatment modality and perioperative outcomes. Models were adjusted for baseline characteristics, including age, sex, diabetes mellitus, chronic kidney disease, hypertension, chronic heart failure, COPD, cerebrovascular disease, year of surgery and obesity. Logistic regression results are presented as odds ratios (ORs) with 95% confidence intervals (CIs). Statistical significance was defined as two-sided *p* < 0.05.

## 3. Results

### 3.1. Patient Characteristics

From 2005 to 2023, 53,427 patients underwent surgical management of UTUC. The majority were treated with radical nephroureterectomy (RNU; n = 41,289, 77.3%), followed by endoscopic laser destruction (n = 7391, 13.8%) and SUR (n = 4747, 8.9%). Median age was 73 years (IQR 66–79), and 66% were male. Patients undergoing endoscopic procedures were older (median 76 years vs. 73 years for RNU and SUR, *p* < 0.001) and had a higher prevalence of chronic kidney disease (30% vs. 26%, *p* < 0.001). Common comorbidities included hypertension (60%) and diabetes (20%). Overall complication rates included sepsis (1.7%), acute kidney disease (6.6%), transfusion (21%), and in-hospital mortality (1.7%) (Table 1).

### 3.2. Trends and Perioperative Outcomes by Treatment Modality

**Figure 1:**
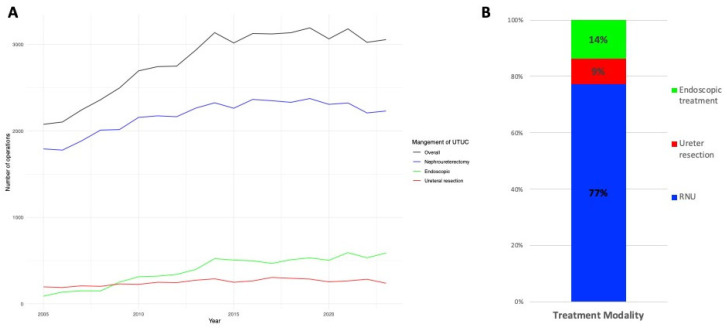
Trends in surgical treatment modalities for UTUC (2005–2023). (**A**) Annual case counts stratified by treatment modality. (**B**) Relative proportions of each treatment modality across the full study period.

Treatment patterns evolved considerably during the study period (Figure 1). In 2005, RNU accounted for more than 85% of procedures, while endoscopic laser destruction represented fewer than 10%. By 2023, the proportion of RNU had declined to approximately 70%, accompanied by a steady rise in endoscopic laser destruction to nearly 20%. SUR remained stable at around 8–10% throughout the period.

Perioperative morbidity varied by modality. RNU was associated with the highest rates of transfusion (24%), acute kidney disease (7.3%), and ICU admission (20%), with in-hospital mortality of 1.9%. Endoscopic laser destruction, increasingly performed in elderly and comorbid patients, demonstrated the most favorable short-term profile, with a median hospital stay of 3 days, mortality of 0.5%, and transfusion in 4.0% of cases. Segmental ureteral resection showed intermediate morbidity but carried elevated risks of embolism (2.6%) and ileus (3.4%) (Table 2).

### 3.3. Trends and Perioperative Outcomes with Lymphadenectomy

**Figure 2:**
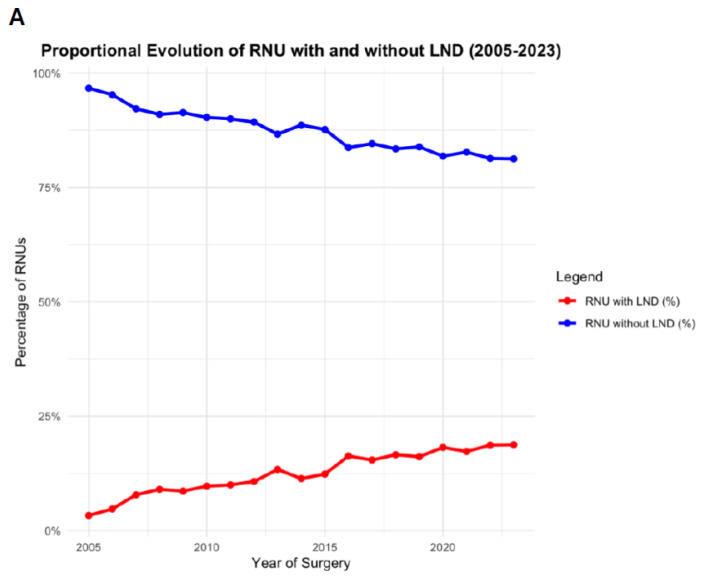

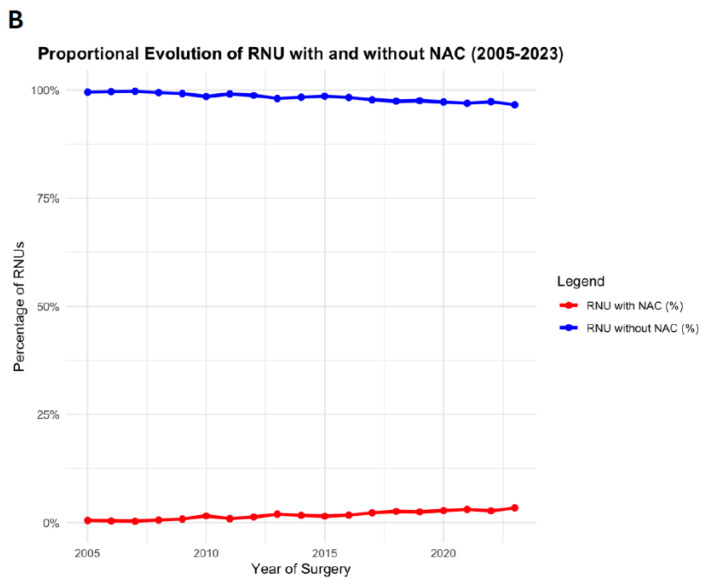
Trends in lymphadenectomy (**A**) and neoadjuvant chemotherapy (**B**) use during RNU for UTUC (2005–2023). Legend: Proportion of RNU patients receiving lymphadenectomy or neoadjuvant chemotherapy over time.

Among RNU patients, 5314 (13.0%) underwent concomitant lymphadenectomy (Supplementary Table S1). Patients receiving lymphadenectomy were slightly younger (median 72 vs. 73 years, *p* < 0.001) and had COPD less frequently (8.8% vs. 10%, *p* < 0.001). Compared with RNU without lymphadenectomy, perioperative morbidity was higher, including transfusion (28% vs. 24%, *p* < 0.001), acute kidney disease (9.2% vs. 7.0%, *p* < 0.001), ileus (3.1% vs. 2.5%, *p* = 0.010), and urinoma (1.3% vs. 0.8%, *p* < 0.001), while in-hospital mortality was similar (2.0% vs. 1.9%). In multivariable analysis, lymphadenectomy was associated with increased risks of transfusion (OR 1.47, 95% CI 1.37–1.57), AKD (OR 1.19, 95% CI 1.07–1.32), ICU admission (OR 1.21, 95% CI 1.13–1.30), and urinoma (OR 1.58, 95% CI 1.20–2.06) (Table 3). Utilization of lymphadenectomy increased during the study period, rising from 1.1% of RNU cases in 2005 to more than 5% by 2013, with a continued upward trajectory through 2023 (Figure 2).

### 3.4. Trends and Perioperative Outcomes with Neoadjuvant Chemotherapy

A total of 726 RNU patients (1.7%) received neoadjuvant chemotherapy (Supplementary Table S2). These patients were younger (median 70 vs. 73 years, *p* < 0.001), had chronic kidney disease more frequently (33% vs. 26%, *p* < 0.001), and were more likely to undergo lymphadenectomy (19% vs. 13%, *p* < 0.001). NAC was not associated with higher in-hospital mortality (1.7% vs. 1.9%, *p* = 0.670) but was associated with increased rates of ileus (3.9% vs. 2.6%, *p* = 0.046) and urinoma (2.1% vs. 0.8%, *p* < 0.001). On multivariable analysis, NAC was linked to higher risks of transfusion (OR 1.28, 95% CI 1.07–1.52) and urinoma (OR 2.31, 95% CI 1.31–3.78), with no significant associations for sepsis, acute kidney disease, or mortality (Table 4). Use of NAC increased steadily, from approximately 0.5% of RNU cases in 2005 to 1.5% in 2015, with further expansion to 3.4% by 2023 (Figure 2).

## 4. Discussion

Over the past decade, UTUC has been recognized as a distinct tumor entity apart from urothelial carcinomas of the lower urinary tract [12]. Correspondingly, the European Association of Urology (EAU) and the American Association of Urology (AUA) have implemented UTUC-specific therapy guidelines in 2011 and 2023 to standardize clinical management and improve patient outcomes [10,13]. Technical advances in endourology and new insights into the efficacy of lymphadenectomy and neoadjuvant chemotherapy have shaped the therapeutic landscape of UTUCs. Current literature has largely focused on epidemiological trends and the adoption of novel surgical techniques, particularly the increasing use of laparoscopic and robotic-assisted radical nephroureterectomy [14,15]. In the era of individualized medicine, however, the greater clinical challenge lies in integrating multimodal treatment strategies and organ-sparing approaches in appropriately selected patients to balance oncological control with preservation of function and quality of life.

Nevertheless, it remains uncertain to what extent clinical management follows guideline recommendations and implements these evolving treatment strategies in the real-world setting of this rare tumor entity. Using a nationwide cohort of more than 50,000 patients over a 19-year period, the present study characterizes contemporary treatment patterns with particular focus on organ-sparing approaches, the comorbidity profile of treated patients, and the real-world utilization of lymph node dissection and neoadjuvant chemotherapy. This large population-based dataset provides novel insights into how multimodal treatment concepts are implemented in routine clinical practice and their associated perioperative risks.

This nationwide cohort study demonstrates that older and frail patients were more likely to undergo endoscopic laser destruction, while radical nephroureterectomy (RNU) remains the predominant treatment approach. Given that UTUC primarily affects elderly patients who often have limited life expectancy and significant comorbidities, organ-sparing strategies such as endoscopic laser therapy represent a reasonable and clinically valuable option for appropriately selected, fragile patients [16]. Additionally, evidence for laser ablation has increased over the past decade, and new laser techniques make tumor ablation more efficient [17,18,19]. Also, laser ablation has been used in selected cases for high-grade UTUC and showed comparable outcomes to RNU [20]. Therefore, endoscopic laser ablation plays a promising role in kidney-sparing approaches for UTUC.

Over the study period, the use of SUR did not increase, despite growing evidence of comparable oncologic outcomes and superior postoperative renal function [21,22]. Patients undergoing SUR showed similar demographic characteristics and comorbidities to those treated with radical nephroureterectomy (RNU), suggesting that tumor location rather than patient frailty primarily guides surgical decision-making within the concept of kidney-sparing surgery (KSS). However, the indication for RNU can be very wide, from extensive low-grade UTUC to small high-grade UTUC and even locally advanced or metastatic UTUC [23,24]. Therefore, comparison of complications after RNU with those after SUR must be interpreted cautiously. In general, data on postoperative complications after SUR remain extremely limited. A small cohort study reported fewer complications following SUR compared with RNU [25], whereas our analysis demonstrated higher rates of embolism and ileus in the SUR cohort, while RNU was associated with longer ICU stays and higher transfusion rates. Postoperative mortality did not differ between groups. Overall, SUR does not appear to play a major role within KSS, whereas endoscopic laser surgery is emerging as the predominant nephron-sparing approach to preserve renal function in the era of personalized medicine.

While our study supports prior findings of trends in treatment modalities, our findings extend prior database studies on the use of LNDs and the use of neoadjuvant chemotherapy. While LNDs are still performed in a minority of patients, they have gained increasing relevance over time. The proportion of RNU cases with LND rose steadily from 3% in 2005 to more than 19% of all RNU in 2023. To date, the oncological benefits of LND are still controversially discussed, but a meta-analysis reported improved outcomes in high-risk patients [26,27]. However, performing LND improves staging and could help identify high-risk patients who would benefit from adjuvant therapy [28]. Although the EAU guidelines consider LND to carry only a minimal risk of major complications, this is based on a limited study cohort, and our large data analysis underlines the association of increased risk for complications in the real-world setting [29]. As the coding of lymph node dissection does not capture the precise extent of dissection, the dataset may not fully reflect the potential impact of dissection extent on perioperative risk. Accordingly, LND should be selectively performed in high-risk UTUC patients at the time of RNU, balancing its potential oncologic benefit against the increased perioperative morbidity and ensuring meticulous perioperative management.

Similarly, NAC use expanded during the study period and reached more than 3% of RNU cases in 2023. Efficacy of NAC in UTUC has been well reported for different regimens, and considering a relevant decrease in renal function after RNU, NAC plays a pivotal role [7,8]. First insights have shown that NAC for UTUC is mostly administered at academic centers [30], so further implementation to identify NAC patients is necessary to ensure the best patient care in the real-world setting. The low proportion of patients receiving NAC may also be related to the advanced age, frailty, and significant comorbidity burden frequently observed in patients with UTUC, which may limit eligibility for systemic therapy. While NAC provides oncological benefit, it has been linked to higher rates of urinoma and transfusion in our dataset. Importantly, no prospective trials have compared adjuvant versus neoadjuvant chemotherapy in UTUC, and no phase III NAC trial has been reported, leaving guideline recommendations limited.

This study has limitations inherent to retrospective analyses of administrative data. Coding inaccuracies and the absence of detailed oncologic variables (e.g., tumor stage, grade, surgical margin status) or long-term outcomes restricted risk stratification and prognostic assessment. Consequently, based on our results, we cannot draw a direct conclusion that risk-adapted therapy or individualized treatment regimens were performed. Accordingly, results must be interpreted carefully because patients receiving LND and NAC are probably of higher tumor stage, present more complex surgeries and might be at a different performance status. In addition, because the database captures only inpatient hospitalizations, neoadjuvant chemotherapy administered in outpatient settings may not be fully recorded, potentially leading to an underestimation of the true NAC utilization rate. Outpatient procedures, postdischarge events, and hospital- or surgeon-level characteristics were not captured, and patient or surgeon preferences influencing treatment selection remain unknown. Furthermore, perioperative complications were identified using standardized ICD and OPS codes from administrative data, and independent chart validation was not feasible; therefore, coding inaccuracies cannot be entirely excluded.

Despite these constraints, the present analysis displays the largest nationwide UTUC cohort reported to date, with the longest investigated period, and uniquely evaluates real-world utilization and perioperative outcomes of lymphadenectomy and neoadjuvant chemotherapy. These data illustrate that real-world clinical practice in Germany has gradually integrated guideline-driven multimodal strategies into the management of this rare disease.

## 5. Conclusions

This nationwide study demonstrates evolving treatment patterns in UTUC. Endoscopic laser destruction has become widely adopted, particularly in elderly and comorbid patients, while guideline-driven lymphadenectomy is increasingly implemented during RNU. In contrast, neoadjuvant chemotherapy remains underutilized, underscoring the need for greater awareness and education to optimize multimodal care.

## Figures and Tables

**Table 1:** Baseline patient characteristics by treatment modality.

Characteristic	Overall (n = 53,427)	RNU (n = 41,289)	Endoscopic Laser Destruction (n = 7391)	Segmental Ureter Resection (n = 4747)	*p*-Value
Sex (male)	35,385 (66%)	26,941 (65%)	5105 (69%)	3339 (70%)	<0.001
Age (IQR)	73.0 (66.0–79.0)	73.0 (65.0–79.0)	76.0 (68.0–82.0)	73.0 (66.0–79.0)	<0.001
LOS (IQR)	11.0 (8.0–16.0)	12.0 (9.0–17.0)	3.0 (2.0–5.0)	13.0 (9.0–18.0)	<0.001
**Comorbidities**					
Diabetes	10,495 (20%)	7927 (19%)	1648 (22%)	920 (19%)	<0.001
Chronic Heart Failure	4123 (7.7%)	3147 (7.6%)	600 (8.1%)	376 (7.9%)	0.290
CKD (Chronic Kidney Disease)	14,241 (27%)	10,870 (26%)	2198 (30%)	1173 (25%)	<0.001
COPD	5398 (10%)	4222 (10%)	721 (9.8%)	455 (9.6%)	0.220
Cerebrovascular Disease	1394 (2.6%)	1100 (2.7%)	176 (2.4%)	118 (2.5%)	0.320
Hypertension	32,243 (60%)	24,900 (60%)	4540 (61%)	2803 (59%)	0.031
Obesity	3923 (7.3%)	3156 (7.6%)	409 (5.5%)	358 (7.5%)	<0.001
**Complications**					
Sepsis	898 (1.7%)	720 (1.7%)	76 (1.0%)	102 (2.1%)	<0.001
AKD (acute kidney disease)	3501 (6.6%)	3010 (7.3%)	213 (2.9%)	278 (5.9%)	<0.001
Embolism	418 (0.8%)	273 (0.7%)	21 (0.3%)	124 (2.6%)	<0.001
Transfusion	11,234 (21%)	10,091 (24%)	298 (4.0%)	845 (18%)	<0.001
Mortality	904 (1.7%)	790 (1.9%)	36 (0.5%)	78 (1.6%)	<0.001
Urinoma	404 (0.8%)	348 (0.8%)	12 (0.2%)	44 (0.9%)	<0.001
Ileus	1252 (2.3%)	1080 (2.6%)	12 (0.2%)	160 (3.4%)	<0.001
ICU admission	8889 (17%)	8146 (20%)	103 (1.4%)	640 (13%)	<0.001
**Age groups**					<0.001
<50	1247 (2.3%)	1019 (2.5%)	106 (1.4%)	122 (2.6%)	
50–59	4582 (8.6%)	3733 (9.0%)	454 (6.1%)	395 (8.3%)	
60–69	11,929 (22%)	9543 (23%)	1319 (18%)	1067 (22%)	
70–79	20,599 (39%)	16,270 (39%)	2513 (34%)	1816 (38%)	
>80	15,070 (28%)	10,724 (26%)	2999 (41%)	1347 (28%)	

Legend: Demographic, comorbidity, and perioperative data of patients treated with radical nephroureterectomy (RNU), endoscopic laser destruction, or segmental ureteral resection.

**Table 2:** Perioperative complications by treatment modality.

	OR	95% CI	*p*-Value
**Transfusion**			
RNU (Ref.)			
URS	0.11	0.10, 0.12	<0.001
Ureteral Segment Resection	0.66	0.61, 0.71	<0.001
**Sepsis**			
RNU (Ref.)			
URS	0.54	0.42, 0.68	<0.001
Ureteral Segment Resection	1.22	0.98, 1.50	0.065
**Mortality**			
RNU (Ref.)			
URS	0.21	0.15, 0.29	<0.001
Ureteral Segment Resection	0.82	0.64, 1.03	0.100
**Acute Kidney Disease**			
RNU (Ref.)			
URS	0.28	0.24, 0.32	<0.001
Ureteral Segment Resection	0.75	0.66, 0.85	<0.001
**Embolism**			
RNU (Ref.)			
URS	0.38	0.24, 0.58	<0.001
Ureteral Segment Resection	3.92	3.15, 4.85	<0.001
**Ileus**			
RNU (Ref.)			
URS	0.06	0.03, 0.09	<0.001
Ureteral Segment Resection	1.28	1.08, 1.51	0.005
**ICU admission**			
RNU (Ref.)			
URS	0.06	0.05, 0.07	<0.001
Ureteral Segment Resection	0.63	0.58, 0.69	<0.001
**Urinoma**			
RNU (Ref.)			
URS	0.18	0.10, 0.31	<0.001
Ureteral Segment Resection	1.09	0.78, 1.48	0.600
**Length of hospital stay**			
RNU			
URS	−9.40	−9.6, −9.2	<0.001
Ureteral Segment Resection	1.20	0.92, 1.5	<0.001

Legend: Complication rates across treatment modalities adjusted for baseline characteristics, including age, sex and comorbidities. RNU was the reference for odds ratios.

**Table 3:** Risk of complications associated with lymphadenectomy in RNU patients.

	OR	95% CI	*p*-Value
No Lymphadenectomy as Reference			
Transfusion	1.47	1.37, 1.57	<0.001
Sepsis	1.10	0.88, 1.36	0.400
Mortality	1.22	0.98, 1.50	0.064
Acute Kidney Disease	1.19	1.07, 1.32	<0.001
Embolism	0.99	0.70, 1.38	>0.900
Ileus	1.22	1.03, 1.45	0.019
ICU admission	1.21	1.13, 1.30	<0.001
Urinoma	1.58	1.20, 2.06	<0.001
Length of hospital stay	0.76	0.49, 1.0	<0.001

Legend: Univariate analysis showing odds ratios for perioperative complications with lymphadenectomy vs. no lymphadenectomy.

**Table 4:** Risk of complications associated with neoadjuvant chemotherapy in RNU patients.

	OR	95% CI	*p*-Value
No Neoadjuvant Chemotherapy as Reference			
Transfusion	1.28	1.07, 1.52	0.005
Sepsis	1.08	0.58, 1.81	0.800
Mortality	1.04	0.55, 1.79	0.900
Acute Kidney Disease	1.02	0.78, 1.33	0.900
Embolism	1.77	0.87, 3.18	0.079
Ileus	1.45	0.96, 2.09	0.059
ICU admission	0.92	0.75, 1.11	0.400
Urinoma	2.31	1.31, 3.78	0.002
Length of hospital stay	0.36	−0.32, 1.0	0.300

Legend: Univariate analysis showing odds ratios for perioperative complications with neoadjuvant chemotherapy vs. no chemotherapy.

## Data Availability

The data presented in this study are available upon request from the corresponding author.

## Supplementary Materials

The following supporting information can be downloaded at: https://www.mdpi.com/article/10.3390/jcm15072536/s1, Table S1: Baseline characteristics of RNU patients by lymphadenectomy status; Table S2: Baseline characteristics of RNU patients by receipt of neoadjuvant chemotherapy.
