# Supplementary material for: Trends in Treatment and Perioperative Outcomes of Upper Tract Urothelial Carcinoma: The Evolving Role of Lymphadenectomy and Neoadjuvant Chemotherapy
Source: J Clin Med. 2026 Mar 26;15(7):2536. doi: 10.3390/jcm15072536 (PMC13074189; doi:10.3390/jcm15072536)
Supplement: Supplementary file 1 [file jcm-15-02536-s001.zip › jcm-4174610-supplementary.pdf]

Supplementary Table S1. Baseline characteristics of RNU patients by lymphadenectomy status

Characteristic	All RNU N = 41,289	RNU without LND N = 35,975	RNU with LND N = 5,314	p-value
Sex	26,941 (65%)	23,468 (65%)	3,473 (65%)	0.870
Age	73.0 (65.0-79.0)	73.0 (65.0-79.0)	72.0 (64.0-78.0)	<0.001
LOS (d)	12.0 (9.0-17.0)	12.0 (9.0-17.0)	11.0 (9.0-16.0)	<0.001
Diabetes Mellitus	7,927 (19%)	6,987 (19%)	940 (18%)	0.003
Chronic Heart Failure	3,147 (7.6%)	2,822 (7.8%)	325 (6.1%)	<0.001
Chronic Kidney Disease	10,870 (26%)	9,459 (26%)	1,411 (27%)	0.700
COPD	4,222 (10%)	3,753 (10%)	469 (8.8%)	<0.001
Cerebrovascular disease	1,100 (2.7%)	980 (2.7%)	120 (2.3%)	0.054
Hypertension	24,900 (60%)	21,820 (61%)	3,080 (58%)	<0.001
Obesity	3,156 (7.6%)	2,759 (7.7%)	397 (7.5%)	0.630
Sepsis	720 (1.7%)	625 (1.7%)	95 (1.8%)	0.840
Acute Kidney Disease	3,010 (7.3%)	2,521 (7.0%)	489 (9.2%)	<0.001
Embolism	273 (0.7%)	233 (0.6%)	40 (0.8%)	0.430
Transfusion	10,091 (24%)	8,605 (24%)	1,486 (28%)	<0.001
Mortality	790 (1.9%)	684 (1.9%)	106 (2.0%)	0.680
Urinoma	348 (0.8%)	279 (0.8%)	69 (1.3%)	<0.001
Ileus	1,080 (2.6%)	913 (2.5%)	167 (3.1%)	0.010
Use of NAC	726 (1.8%)	587 (1.6%)	139 (2.6%)	<0.001
Stay at ICU	8,146 (20%)	7,048 (20%)	1,098 (21%)	0.070
Age group				<0.001
<50	1,019 (2.5%)	866 (2.4%)	153 (2.9%)	
50-59	3,733 (9.0%)	3,171 (8.8%)	562 (11%)	
60-69	9,543 (23%)	8,177 (23%)	1,366 (26%)	
70-79	16,270 (39%)	14,206 (39%)	2,064 (39%)	
>80	10,724 (26%)	9,555 (27%)	1,169 (22%)	

Legend: radical nephroureterectomy (RNU), Neoadjuvant Chemotherapy (NAC), Length of stay (LOS), Chronic obstructive pulmonary disease (COPD), Intensive care unit (ICU)

Supplementary Table S2. Baseline characteristics of RNU patients by receipt of neoadjuvant chemotherapy

Characteristic	All RNU<br>N = 41,289	RNU without NAC<br>N = 40,563	RNU with NAC<br>N = 726	p-value
Sex	26,941 (65%)	26,499 (65%)	442 (61%)	0.014
Age	73.0 (65.0-79.0)	73.0 (65.0-79.0)	70.0 (63.0-76.0)	<0.001
LOS (d)	12.0 (9.0-17.0)	12.0 (9.0-17.0)	11.0 (8.0-16.0)	<0.001
Diabetes Mellitus	7,927 (19%)	7,809 (19%)	118 (16%)	0.047
Chronic Heart Failure	3,147 (7.6%)	3,098 (7.6%)	49 (6.7%)	0.410
Chronic Kidney Disease	10,870 (26%)	10,634 (26%)	236 (33%)	<0.001
COPD	4,222 (10%)	4,157 (10%)	65 (9.0%)	0.280
Cerebrovascular Disease	1,100 (2.7%)	1,090 (2.7%)	10 (1.4%)	0.040
Hypertension	24,900 (60%)	24,475 (60%)	425 (59%)	0.350
Obesity	3,156 (7.6%)	3,105 (7.7%)	51 (7.0%)	0.570
Sepsis	720 (1.7%)	707 (1.7%)	13 (1.8%)	>0.990
Acute Kidney Disease	3,010 (7.3%)	2,945 (7.3%)	65 (9.0%)	0.100
Embolism	273 (0.7%)	263 (0.6%)	10 (1.4%)	0.030
Transfusion	10,091 (24%)	9,904 (24%)	187 (26%)	0.430
Mortality	790 (1.9%)	778 (1.9%)	12 (1.7%)	0.670
Urinoma	348 (0.8%)	333 (0.8%)	15 (2.1%)	<0.001
Ileus	1,080 (2.6%)	1,052 (2.6%)	28 (3.9%)	0.046
Lymphadenectomy	5,314 (13%)	5,175 (13%)	139 (19%)	<0.001
Stay at ICU	8,146 (20%)	8,026 (20%)	120 (17%)	0.032
Age group				<0.001
<50	1,019 (2.5%)	998 (2.5%)	21 (2.9%)	
50-59	3,733 (9.0%)	3,661 (9.0%)	72 (9.9%)	
60-69	9,543 (23%)	9,324 (23%)	219 (30%)	
70-79	16,270 (39%)	15,980 (39%)	290 (40%)	
>80	10,724 (26%)	10,600 (26%)	124 (17%)	

Legend: radical nephroureterectomy (RNU), Neoadjuvant Chemotherapy (NAC), Length of stay (LOS), Chronic obstructive pulmonary disease (COPD), Intensive care unit (ICU)
